# Supplementary material for: Correlation between Anthropometric and Ultrasound Measurement for Dry Needling of the Iliocostalis Lumborum Muscle with a Safety Protocol: A Cross-Sectional Observational Study
Source: Healthcare (Basel). 2022 Dec 7;10(12):2470. doi: 10.3390/healthcare10122470 (PMC9777974; doi:10.3390/healthcare10122470)
Supplement: Supplementary file 1 [file healthcare-10-02470-s001.zip › healthcare-1941317-supplementary.pdf]

Annexes of the study of "Correlation of anthropometric measurements and ultrasound measurements to establish dry needling protocol of the iliocostalis lumborum muscle: cross-sectional observational study."

Annex SI. Normality tests.

Table S1. Normality test

#	W	p value
Weight	0.057	0.845
Height	0.068	0.597
Body Mass Index	0.059	0.813
Neutral axillary circumference	0.093	0.156
Neutral xiphoid circumference	0.111	0.038
Neutral abdominal circumference	0.110	0.039
Inspiration axillary circumference	0.085	0.253
Axillary expiratory circumference	0.100	0.088
Inspirational xiphoid circumference	0.125	0.010
Expiratory xiphoid circumference	0.092	0.159
Inspiration abdominal circumference	0.099	0.094
Abdominal expiratory circumference	0.091	0.179

#	W	p value
Chest-triceps skinfolds thickness	0.095	0.137
Abdomen-iliac crest skinfolds thickness	0.180	0.000
Thigh skinfolds thickness	0.086	0.241
fat percentage	0.080	0.354
Right L2 without compression: skin-lumbar iliocostal upper border	0.292	0.000
Right L2 without compression: lumbar iliocostal skin-inferior border	0.325	0.000
Right L2 without compression: skin-kidney	0.328	0.000
Right L2 with compression: skin-lumbar iliocostal upper border	0.303	0.000
Right L2 with compression: lumbar iliocostal skin-lower edge	0.335	0.000
Right L2 with compression: skin-kidney	0.333	0.000
Left L2 without compression: skin-lumbar iliocostal upper border	0.274	0.000
Left L2 without compression: lumbar iliocostal skin-inferior border	0.309	0.000
Left L2 without compression: skin-kidney	0.320	0.000
Left L2 with compression: skin-lumbar iliocostal upper border	0.306	0.000
Left L2 with compression: lumbar iliocostal skin-lower edge	0.333	0.000

#	W	p value
Left L2 with compression: skin-kidney	0.334	0.000
Right L4 without compression: skin-lumbar iliocostal upper border	0.278	0.000
Right L4 without compression: lumbar iliocostal skin-inferior border	0.309	0.000
Right L4 without compression: skin-peritoneum	0.308	0.000
Right L4 with compression: skin-lumbar iliocostal upper border	0.300	0.000
Right L4 with compression: lumbar iliocostal skin-inferior border	0.316	0.000
Right L4 with compression: skin-peritoneum	0.320	0.000
Left L4 without compression: skin-lumbar iliocostal upper border	0.281	0.000
Left L4 without compression: lumbar iliocostal skin-inferior border	0.324	0.000
Left L4 without compression: skin-peritoneum	0.327	0.000
Left L4 with compression: skin-lumbar iliocostal upper border	0.314	0.000
Left L4 with compression: lumbar iliocostal skin-inferior border	0.329	0.000
Left L4 with compression: skin-peritoneum	0.331	0.000

Annex SII. Results depending on kind of measurements (with compression VS without compression).

Table S2. Results depending on kind of measurements (with VS without compression).

#	P hdvxuhp hqwv# z lwkrxw#frp suhvvlrq#	P hdvxuhp hqwv# z lwk#frp suhvvlrq#	[d]s#ydoxh#
q#	99#	99#	#
O5#uljkw#vnlq(nlgqh|#	57169Ø5613;#	591:9Ø571<8#	313;:#
O5#dhiw#vnlq(nlgqh|#	57144Ø55189#	5818;Ø561;8#	3178#
O7#uljkw#vnlq(shulwrqhxp #	5:179Ø591:;#	6314;Ø5;1;3#	31578#
O7#dhiw#vnlq(shulwrqhxp #	5;18<Ø5:16:#	631430Ø5;195#	31987#

G dwd#h{suhvvhg#dv#p hdqØ#vwdqgdug#ghyldwlrq1

[d#]vljqlilfdqw#li#s?31381#

Annex SIII. Regression analysis. Dominant vs. non-dominant side comparison.

Table S3. Dominant vs. non-dominant side comparison.

#	grp lqdqw#dhj#	qrq(grp lqdqw#dhj#	dw#s#ydoxh#
q#	9;#	9;#	#
O5#z lwkrxw#vnlq;frp suhvvlrq#xsshu#ordfrvwdo#xp edu#hgjh#	;184Ø<148#	:1k4Ø;17;#	318#
O5#z lwkrxw#vnlq#frp suhvvlrq#lqihulru#ordfrvwdo#xp edu#hgjh#	4;1:;Ø4:167#	4<15<Ø4:1::#	31;97#
O5#z lwkrxw#nlgqh|#vnlq#frp suhvvlrq#	58145Ø56156#	58156Ø56143#	31;4;#
O5#z lwk#xp edu#ordfrvwdo#xsshu#hgjh#vnlq#frp suhvvlrq#	;17<Ø;1:5#	;1:6Ø<148#	31::4#
O5#z lwk#xp edu#ordfrvwdo#orz hu#hgjh#vnlq#frp suhvvlrq#	54145Ø4<17<#	531;:Ø4<14<#	31:57#
O5#z lwk#nlgqh|#vnlq#frp suhvvlrq#	5:173Ø571k3#	591:7Ø57167#	31964#
O7#z lwkrxw#vnlq#frp suhvvlrq#xsshu#ordfrvwdo#xp edu#hgjh#	441;3Ø45184#	<1:9Ø44136#	31456#
O7#z lwkrxw#vnlq#frp suhvvlrq#lqihulru#ordfrvwdo#xp edu#hgjh#	55197Ø5417:#	55135Ø54187#	31k;<#
O7#z lwkrxw#frp suhvvlrq#vnlq#shulwrqhxp #	5;17;Ø591k<#	5;177Ø5:158#	31;66#
O7#z lwk#vnlq#frp suhvvlrq#xsshu#ordfrvwdo#xp edu#hgjh#	431;8Ø441k3#	<1;7Ø4317;#	31958#
O7#z lwk#vnlq#frp suhvvlrq#orz hu#ordfrvwdo#xp edu#hgjh#	5714<Ø56143#	5616:Ø551:9#	31976#
O7#z lwk#shulwrqhxp #vnlq#frp suhvvlrq#	631k5Ø5<135#	5<1:;Ø5;16;#	3196;#

Gdwd#h{suhvvhg#dv#p hdqØ#vwdqgdug#ghyldwlrq1#

grp lqdqw#dnj# qrq(grp lqdqw#dnj# [dw]s#ydoxh#

#

[d]vljqlilfdqw#li#s?31381#

Annex SIV. Smoothed model with the dependent variable L4 without compression

Table S4. Smoothed model with the dependent variable L4 without compression.

#	HGI#	gi$_{uhi}$#	I#	[d]s# ydoxh#
Djh#	31333#	6#	31333#	31k;:#
Erg|#Pdvv#Lqgh{#	4174<#	5#	5147:#	3137;#
Qhxwudo#d{lodu|#flufxp ihuhqfh#	31;6<#	6#	41558#	31366#
Qhxwudo#degrp lqdo#flufxp ihuhqfh#	41k:8#	6#	;1:59#	?31334#
Fkhvw0wulsfhsv#vnlqirog#wklfnqhvv#	4178;#	6#	51745#	31345#
Degrp hq0loldf# fuhw# vnlqirog# wklfnqhvv#	41333#	6#	71k8:#	?31334#
Wkljk#vnlqirog#wklfnqhvv#	41588#	6#	31k46#	31445#

HGI=#Hiihfwlyh#ghjuhhv#ri#iuhhgrp1#gi$_{uhi}$=
uhihuhqfh#ghjuhhv#ri#iuhhgrp#

[d]vljqlilfdqw#s?3138:#

Annex SV. Smoothed model with the dependent variable L4 with compression.

Table S5. Smoothed model with the dependent variable L4 with compression.

#	HGI#	gi	I#	[d]s0 ydoxh#
Djh#	31333#	6#	31333#	31k77#
Erg|#P dvv#Lqgh{#	41k3:#	6#	51;:6#	3133<#
Qhxwudo#d{ldu|#flufxp ihuhqfh#	318:8#	6#	318<<#	313:6#
Qhxwudo#degrp lqdo#flufxp ihuhqfh#	41k<:#	6#	431593#	?31334#
Fkhvw0wulfhsv#vnlqirog#wklfnqhvv#	31;<5#	6#	519;7#	31336#
Degrp hq(ldf# fuhw# vnlqirog# wklfnqhvv#	31k96#	6#	71937#	?31334#
Wkljk#vnlqirog#wklfnqhvv#	31333#	6#	31333#	318:5#

HGI=#Hiihfwlyh#ghjuhhv#ri#iuhhgrp 1#

gi_{uhi}=#uhihuhqfh#ghjuhhv#ri#iuhhgrp #

[d]vljqlilfdqw#s?31384#

Annex SVI. Smoothed model with the dependent variable L2 without compression.

Table S6. Smoothed model with the dependent variable L2 without compression.

#	HGI#	gi$_{uhi}$#	I#	ds; ydoxh#
Djh#	419;5#	6#	;1;87#	?31334#
Erg|#P dvv#Lqgh{#	41686#	6#	41386#	313;7#
Q hxwudo#d{lodu|#flufxp ihuhqfh#	31333#	6#	31333#	3176<#
Q hxwudo#degrp lqdo#flufxp ihuhqfh#	4189;#	6#	41856#	31374#
Fkhvw(wulfhsv#vnlqirog#wklfnqhvv#	513;5#	6#	5;14:5#	?31334#
Degrp hq(lddf# fuhvw# vnlqirog# wklfnqhvv#	31;<5#	6#	451k36#	?31334#
Wkljk#vnlqirog#wklfnqhvv#	31k;;#	6#	<41468#	?31334#

HGI=#Hiihfwlyh#ghjuhhv#ri#iuhhgrp#

gi$_{uhi}$=#Uhihuhqfh#ghjuhhv#ri#iuhhgrp#

[d]vljqlilfdqw#s?3138#

Annex SVII. Smoothed model with the dependent variable L2 with compression.

Table S7. Smoothed model with the dependent variable L2 with compression.

#	HGI#	gi uhi#	I#	ds# ydoxh#
Djh#	31333#	6#	31333#	4#
Erg|#P dvv#Lqgh{#	41674#	5#	41;68#	31397#
Qhxwudo#d{loodu|#flufxp ihuhqfh#	31;96#	6#	418<9#	3134:#
Qhxwudo#degrp lqdo#flufxp ihuhqfh#	41<95#	6#	4314<;#	?31334#
Fkhvw0wulfhsv#vnlqirog#wklfnqhvv#	31;:7#	6#	513:6#	3133<#
Degrp hq0lodf# fuhvw# vnlqirog# wklfnqhvv#	41333#	6#	719::#	?31334#
Wkljk#vnlqirog#wklfnqhvv#	4177:#	6#	4154:#	313;6#

HGI=Hiihfwlyh#ghjuhhv#ri#iuhhgrp #

gi uhi#=#Uhihuhqfh#ghjuhhv#ri#iuhhgrp #

#
dvljqlilfdqw#s?31388#
